# Medication Abortion and Abortion Pill Reversal: An Exploratory Analysis on the Influence of Others in Women’s Decision-Making

**DOI:** 10.7759/cureus.49973

**Published:** 2023-12-05

**Authors:** Katherine A Rafferty, Tessa Longbons

**Affiliations:** 1 Psychology, Iowa State University, Ames, USA; 2 Research, Charlotte Lozier Institute, Arlington, USA

**Keywords:** women's reproductive issues, women's reproductive health, medication abortion, family health communication, health decision-making, abortion pill reversal, abortion

## Abstract

Introduction

As medication abortion accounts for a growing share of abortions in the United States, an increasing number of women are seeking abortion pill reversal (APR). These decisions are typically not made in isolation. However, little research exists on women’s APR decision-making and the role played by people close to them.

Methods

We surveyed women who contacted a national hotline for information on APR and who completed a two-week treatment protocol with progesterone (n = 67). We analyzed women’s open-ended answers using thematic analysis to identify memorable messages about medication abortion and APR. Participants’ communication with other people in their lives was assessed using the Isolation subscale of the Individual Level Abortion Stigma (ILAS) scale, and decision-making difficulty was assessed using a Likert scale.

Results

Thirty-six respondents met the eligibility criteria and filled out the ILAS and decision-difficulty scales. Women tended to talk with family and friends about their medication abortion decisions, while they typically sought information online when deciding about APR. Women reported greater stigma in their disclosures about their abortions than in their disclosures about APR (*p *= 0.006).

Conclusion

Memorable messages influence women’s decisions to pursue medication abortion and APR.

## Introduction

Medication abortion accounts for over half of total abortions in the United States as of 2020 [1]. Medication abortion occurs by taking a combination of two different medications: mifepristone to block progesterone followed by misoprostol to induce contractions and terminate a pregnancy. Approximately 95-99% of women who take both mifepristone and misoprostol terminate their pregnancies [2]. Mifepristone taken alone is not as effective at terminating a pregnancy, with continuing pregnancy rates ranging from 10% to 23.3% [3].

Since 2012, more than 4,000 women have discontinued their medication abortion protocol by not taking misoprostol, and adhering to a progesterone protocol that has allowed them to maintain viable pregnancies [4]. Because mifepristone is a progesterone antagonist, research indicates that the use of supplemental progesterone may block the effects of mifepristone [5]. An ultrasound is used to confirm a viable heart rate for the baby and the placental placement and date of pregnancy. Given these findings, legislators in at least 14 states have passed abortion “reversal” laws that require clinicians to inform a woman during pre-abortion counseling that the medication abortion pills can sometimes be reversed if she changes her mind [6]. Conversely, at least one state has attempted to ban abortion pill reversal (APR) [7].

However, there is currently a dearth of research on women’s medication abortion reversal decision-making. Since important health decisions, like choosing medication abortion or abortion reversal, are typically not made in isolation but rather made within the context of one’s social networks (e.g., family and close friends) [8], it is important to study other people’s influence in women’s medication abortion and reversal decisions. Furthermore, family is an important site for memorable health messages that impact behavior and self-concept [9]. Thus, we initiated the first evidence-based study on medication abortion reversal decision-making to understand the memorable messages from individuals who may likely influence their decisions to seek medication abortion and APR. We focused our analysis on the role of different primary influences (e.g., male partner and those whom the woman is closest with) and the CPM decisions that women make when deciding whether to openly disclose their choice to seek medication abortion and/or APR. We first discussed the history of medication abortion reversal and explained the literature on abortion decision-making. We concluded with a discussion of research on memorable messages and communication privacy theory as frameworks to guide our analysis.

Use of progesterone as medication abortion reversal

The ongoing debate about the use of progesterone for reversing the effects of mifepristone began in 2012 when Drs. Delgado and Davenport reported on six women who took mifepristone and were then administered varying levels of progesterone to attempt to halt their medication abortions [10]. After receiving 200 mg intramuscular progesterone, four of the six women were able to carry their pregnancies to term. A follow-up study completed by Dr. Delgado and colleagues (2018) assessed 754 women who desired to reverse their medication abortion after taking mifepristone, but before taking misoprostol [5]. Findings revealed that the most effective protocols were intramuscular progesterone and high-dose oral progesterone. In addition, there was zero increased risk for birth defects or preterm births. Furthermore, a review of the literature by Dr. Davenport and colleagues (2017) on embryo survival after mifepristone found that the survival rate is between 10% and 23.3% when only exposed to mifepristone at the commonly administered 200 mg dose [3]. Other research on progesterone has confirmed the effective use of progesterone as a treatment for pregnant women who experience recurring miscarriages or bleeding during pregnancy because of low progesterone [11]. In sum, scholars have concluded that progesterone is effective in helping some women maintain viable pregnancies, whether the intent is to reverse a medication abortion or to sustain a desired pregnancy.

However, the use of progesterone for medication abortion reversal is controversial. The American Medical Association and the American College of Obstetricians and Gynecologists have formal declarations stating that there is no scientific evidence supporting its efficacy [12]. Considering these claims, Dr. Creinin et al. (2020) conducted the first double-blind, placebo-controlled, randomized clinical trial of 40 pregnant women who were enrolled at 44-63 days (about two months) of gestation [13]. However, enrollment ceased at 12 women after three participants experienced hemorrhage, so the efficacy of progesterone for mifepristone antagonization could not be ascertained. Nonetheless, among the women who received progesterone, 80% were able to achieve an ongoing pregnancy compared to the 40% of women who received the placebo. Furthermore, in the progesterone group, four of the five women had “gestational cardiac activity” (a fetal heartbeat) at follow-up. Thus, the concerns that have been raised lend credence to the potential benefit of progesterone usage for pregnant women who have taken mifepristone yet desire a medication abortion reversal.

Medication abortion decision-making and role of family and friends

There are multiple, diverse, interrelated reasons women seek abortion that are driven by their unique life circumstances. All of these reasons are personal and interrelated, demonstrating the complexity of women’s medication abortion decision-making and the influence of other people in this decision [14]. A recent analysis by Rafferty and Longbons (2021) found that women who anonymously shared their medication abortion narratives on a public blogging website reported feeling both unprepared and knowledgeable prior to choosing a medication abortion and struggled with silence and openness when attempting to talk about their medication abortion with other people [15]. Yet, a dearth of research remains on women’s use of APR and how, if at all, others may influence their decisions to seek APR after beginning the medication abortion protocol. Thus, we sought to study women who have self-reported choosing to seek medication abortion followed by APR.

A previous analysis of this same dataset found that women who had undergone the APR process scored their communication with their APR providers as significantly better than their communication with their abortion providers [16]. We also found that women considered their abortion decisions to have been significantly more difficult than their decisions to pursue APR. The goal of the present study is to understand the different messages and people within a woman’s support network who are likely to influence her decision to seek medication abortion followed by medication abortion reversal. As previously mentioned, important health decisions like choosing medication abortion or abortion reversal are typically not made in isolation, but rather these decisions are normally made within the context of one’s social networks (e.g., family and close friends) [8]. Family and friends have a significant role in shaping health behavior and influencing health decisions, and these memorable messages are often regarded as impacting one’s self-concept and behavior [9]. Barriers and facilitators related to seeking abortion services are dependent upon other people, most notably support from family and friends and the man involved in the pregnancy (or lack thereof) [17]. Pressure to seek abortion from parents or partners and fear of an unsupportive reaction by family and friends are both associated with being less confident about one’s abortion decision and having poorer coping post-abortion [18]. A lack of personal autonomy in the abortion decision-making process and limited emotional support from others are two significant factors that contribute to women’s feelings of regret post-abortion [18].

The paucity of research on medication abortion decision-making [14,15] and the complete absence of research on women’s abortion reversal decision-making warrant the need for scholars to study these unique women and what influences their decisions for medication abortion and/or reversal. Memorable messages are powerful tools for coping and have been shown to influence individuals’ attitudes and behaviors [9]. Thus, we advance two research questions:

RQ1: What memorable messages influence women’s decisions to seek medication abortion?

RQ2: What memorable messages influence women’s decisions to seek APR?

Communication privacy management theory

A second goal of the study is to understand women’s private information disclosures within their close networks about their medication abortion and APR. Communication privacy management (CPM) theory proffers a suitable framework as it addresses the complexities of managing the ongoing dialectical tension between public information and one’s private information disclosures [19]. The theory is based upon six principles: (a) public-private dialectical tension, (b) conceptualization of private information, (c) privacy rules, (d) shared boundaries, (e) boundary coordination, and (f) boundary turbulence [20]. The first three principles focus on the dialectical tension between public and private information with the principal idea that private information is individually owned, so individuals must decide whether they want to reveal or conceal their private information. Open disclosures are dependent upon a set of five rules that guide one’s boundary management behaviors: culture, gender, context, motivation, and risk-benefit ratio [19]. The second set of CPM principles focuses on the “interaction maxims,” or the ways in which individuals interact with others when revealing and concealing their private information [20]. Individuals who decide to share private information form collective boundaries with other people, and co-owners become equally responsible for managing the owner’s private information [19]. Effective boundary coordination requires the intentional regulation of access and flow of private information [19].

Many scholars have studied private health information disclosures noting that relational quality, reciprocity, family communication patterns, gender differences, and the perceived risk of disclosure are common factors associated with whether individuals choose to disclose their private health information to family and close friends [21]. Research specifically on abortion has found that United States women tend to selectively disclose their abortion to those whom they believe to be supportive [22]. In a more recent analysis, Rafferty and Longbons (2021) found an overarching dialectical tension of silence vs. openness that created difficulties for women to openly talk with others (particularly family members) about their medication abortion [15]. Other research shows that male partners’ qualities and preferences can contribute to difficulty in women’s abortion decisions, while support from partners can improve women’s perceptions of their abortion experiences [17]. Other scholars have found associations between religion and abortion stigma, with Christian women reporting more worry about judgment and more negative feelings of isolation from social networks compared to nonreligious women [23]. In addition, Black women were less worried about abortion stigma compared to White women, and women who had given birth prior to having an abortion scored lower on the worries about judgment subscale compared to women who had never given birth prior to their abortion [23]. In particular, race, religiosity, and education are all associated with significant differences in women’s feelings of isolation about their abortions, or their level of comfort in disclosing to people they are close with [23]. Given this research and the principles of CPM theory [19,20], we propose three research questions to understand women’s disclosures about their medication abortion and APR:

RQ3: How do women’s reported disclosure with people they are close with during the start of the medication abortion compare to their reported disclosure with people they are close with during the medication abortion reversal?

RQ4: How does male partner involvement in the pregnancy influence the medication abortion decision compared to the decision to seek medication abortion reversal?

RQ5: Which demographic factors are associated with more open disclosures to people women are close with?

## Materials and methods

Participants

The sample consisted of 36 women, of whom 35 completely answered the demographic questions. The mean age was 30 (SD = 6.16). Most of the women were White (53%, n = 19), Catholic (25%, n = 9) or another Christian denomination (50%, n = 18), and unmarried (92%, n = 33). Table 1 provides more detailed information about participants.

**Table 1:** Demographics GED: General educational development

Demographics	N	%
Race		
White	19/35	54%
Black	5/35	14%
Asian	1/35	3%
Other	6/35	17%
Multiple	4/35	11%
Mean age	30	
Median age	30	
SD	6.16	
Age (years)		
20-24	6/35	17%
25-29	11/35	31%
30-34	8/35	23%
35-39	8/35	23%
40+	2/35	6%
Education		
Less than high school	2/35	6%
High school/GED	5/35	14%
Some college/tech school	11/35	31%
Degree or finished tech school	13/35	37%
Graduate degree	4/35	11%
Employment Status		
In school	3/35	9%
Employed	21/35	60%
Both	4/35	11%
Neither	7/35	20%
Religion		
Catholic	9/35	26%
Other Christian	18/35	51%
Other Religion	3/35	9%
None: Not religious or spiritual	5/35	14%
Marital Status		
Married	2/35	6%
Divorced	1/35	3%
Single but cohabiting	10/35	29%
Single living alone	22/35	63%
Pregnancy Outcome		
Chose to abort	1/36	3%
Delivered a baby	18/36	50%
Pregnancy loss	6/36	17%
Still pregnant	11/36	31%
Household Income		
Less than $15,000	10/35	29%
$15,000-$29,000	7/35	20%
$30,000-$59,000	7/35	20%
$60,000-$89,000	5/35	14%
$90,000+	6/35	17%
Geographical Region		
South	11/35	31%
West	10/35	29%
Midwest	6/35	17%
Northeast	8/35	23%

Procedure

The online survey was developed with input from subject matter experts and those who work with women who have experienced medication abortion and sought out APR. We also identified reliable and valid measures pertaining to the variables we sought to study. After receiving Iowa State University Institutional Review Board approval (21-459-00) from a large, comprehensive research university in the Midwest, 3,076 women were emailed a recruitment letter with a link to an online Qualtrics survey. These women contacted an organization headquartered in the Midwest between 2018 and 2022 to seek information about medication abortion reversal after taking mifepristone. Women were encouraged to complete the self-administered survey in one sitting, although the survey settings permitted interruptions. These settings allowed women to regulate their own fatigue, emotions, and time management. We sent two follow-up emails at two different time periods over the course of two months and one follow-up text message in the third month to encourage those who had not responded to complete the survey. There were 108 women who started the survey, of whom 67 reported completing the two-week progesterone protocol, meeting our eligibility criterion to finish the survey. Thirty-six women filled out the decision difficulty and abortion stigma scales (54%).

This survey included a combination of open- and closed-ended questions about women’s experiences with taking mifepristone and then seeking a progesterone protocol, in addition to demographic questions. Open-ended questions allowed for a better understanding of the breadth and depth of women’s experiences. Women were asked to consider their experiences with medication abortion and asked, “Please explain why you chose to take mifepristone compared to a surgical abortion. Please describe if there were certain people or messages that influenced this decision. We encourage you to share as much of your story as you are willing to disclose.” Then, women were asked to consider their experiences with APR and asked, “Please explain why you chose to take progesterone to reverse mifepristone. Please describe if there were certain people or messages that influenced this decision. We encourage you to share as much of your story as you are willing to disclose.”

Instruments

Abortion Decision Difficulty Scale

Women reported their perceptions of their decision-making difficulty in choosing to take mifepristone and in choosing to take progesterone for APR using a single-item Likert scale. Women could report that their decision was very difficult, somewhat difficult, neither easy nor difficult, somewhat easy, or very easy.

Telling the People That I Am Closest to

Respondents’ levels of disclosure with people they are close to were measured using the Isolation subscale of the Individual Level Abortion Stigma (ILAS) scale [23]. We chose the Isolation subscale because its six items measure women’s levels of openness and their comfort in discussing their experiences. Responses to each item on the Isolation subscale were measured on four- or five-point Likert scales and then averaged to produce an overall stigma score for each respondent, with higher scores indicating greater levels of stigma and isolation. Women’s responses to this item were analyzed separately and were not used to generate the disclosure scores.

Analysis

To analyze research questions one and two, the first author used thematic analysis [24]. She initially went through all of the women’s open-ended responses to the two questions about the messages that influenced their decision to seek mifepristone, and the messages that influenced their decision to seek APR. She conducted line-by-line coding of these responses to identify common themes across the data. After discussing her findings with the second author, she further revised and developed the codes into larger themes. She then returned to the data to study the relevant themes based on the fit and thoroughness of the different categories. Quotations that were deemed representative were selected and agreed upon by both authors. To answer research questions three to five, differences in women’s decision-making difficulty and level of stigma for abortion versus APR were compared using paired t-tests, while the impact of demographic factors was analyzed using independent samples t-tests.

## Results

For research question one, we analyzed the memorable messages that influenced women’s decisions to seek medication abortion. Most of the women in the study reported talking about their medication abortion decision with their partner, family, friends, and/or medical providers. Each of these women described a memorable message from one of these individuals. Most of the women reported receiving a message that was contrary to their desire for their pregnancy. For example, one woman said: “I chose to take the medication after an hour at the clinic contemplating it. I called my mother beforehand, hoping she’d say no don’t do it. She did at one point but then left it up to me. I even asked the doctor for his opinion, and he said it’s up to you but saying you’re doing the right thing considering the fact I told him my boyfriend didn’t want to.” Several other women shared similar examples where she desired to keep the baby, but close family and friends verbally expressed their desire for her to terminate the pregnancy. In fact, some women reported feeling pressured to have an abortion by their partner, family, or friends. One woman said: “I was pressured into abortion by my ex and his sister, and the provider gave it to me on the spot without time to think. I always wanted to keep her.” Thus, these memorable messages resembled elements of force and quick decision-making, which seemed contrary to women’s desires.

Of the women surveyed who initially desired to have an abortion, these women reported receiving messages from others about the convenience of taking mifepristone over having a surgical abortion. There were three overall themes that characterized the memorable messages received during the medication abortion decision: cost, convenience, and privacy. For instance, one woman shared: “I also wanted it to be more private than in an office with strangers there for the sole purpose of making money while I cried with no support.” Many women reported that their decisions to choose medication abortion were impacted by financial factors (e.g., not being able to afford a child or the cheaper cost of medication abortion over surgical abortion).

The final type of memorable message that women reported receiving was “it’s just a pill.” For example, one woman was told by her provider that “(mifepristone and misoprostol) were okay because it was just a little pill” and compared taking mifepristone to plan B or having a miscarriage. Women were also told by friends that “it is less traumatizing than a surgical abortion.” Some women described the specific nuances of mifepristone and said things like: “I think the main thing that influenced my decision for a medicinal abortion was that I wasn’t comfortable having someone remove parts of a baby out of me. I knew mifepristone would almost be like a miscarriage, so I was more comfortable with that option.” Thus, the phrase “it’s a pill” was either used as a dismissive statement or a phrase to downplay any fears women may have about the abortion process.

To analyze research question two, we studied the memorable messages that influenced women’s decisions to seek APR. Of the women surveyed, most women sought out APR independently, mentioning Google and other online searches as primary ways that they learned about APR. For example, one woman provided a detailed account and said:

I went to Planned Parenthood and took the first dose of a medicinal abortion. When I was leaving the office, I felt a sudden sense of regret and pulled over on the highway. I did some quick googling and came across the APR hotline number and contacted them almost immediately (within 25 minutes of taking the first dose of the medicated abortion). After speaking with a provider, I made the decision to go forth with the progesterone reversal.

Similarly, a different woman shared how she had intentionally distanced herself from all family and friends because they didn’t want the abortion, but she did. After taking the mifepristone at the clinic, she shared:

As soon as I got in my car it was like I snapped back to reality and almost couldn’t believe what just happened. I started driving and before I even got to the highway. I pulled over and started feverishly searching through my phone for a magic UNDO button. And there it was: the APR program. I called them from the side of the road. I didn’t even know where I was but I needed to figure this out. They asked me a ton of questions and told me that the sooner I did something the better the chances are that we would see success. She asked me when I took the pill and I looked at my phone and I said “I don’t know - 15-20 minutes ago?” She immediately walked me through the steps, the chances, the process. I told myself right then and there that this was the right thing for me to do... I got home and I picked up the prescription for the progesterone. Still sitting on my passenger seat were the step 2 pills for the medicated abortion. I felt like I was holding my life in my hands. The devil and the angel…. I sent him an email back letting him know that I made the decision not to go through the abortion. The reversal process was extremely difficult. I was so sick, really pushed my body to its limits and wondered every day if we had made the right choice and if it was even going to work. Now I’m 30 weeks pregnant and thankful for the program and feel so blessed.

Although online sources were the dominant means through which women learned about APR, a few women were told about APR from friends or pregnancy resource centers whom they sought out after taking mifepristone. For example, one woman said: “I chose (APR) because my friends helped me feel supported in moving forward in pregnancy.” Thus, signifying the importance of others in women’s decisions to seek APR.

Finally, women described how they independently arrived at the decision to seek reversal of their medication abortion, rather than because of the influence of outside messages. These women described how their own emotions prompted them to personally seek out information for a way to reverse the effects of mifepristone. Women noted feeling regret, guilt, sadness, and fear after taking mifepristone. For instance, one woman said:

I chose to do this because I felt I couldn’t live with the decision I had just made. I had three children and thought I would be okay going through the abortion, but I wasn’t. I felt empty inside. I felt I was going to be depressed because of this choice.

After seeking information online about APR, women described memorable messages received when talking with medical providers about APR. One woman said:

He assured me that the pills didn’t do anything since I immediately began the progesterone. He reassured me it would be okay, making me more confident about my decision to take the progesterone.

Thus, these messages provided women with the reassurance and confidence to begin the APR process.

To answer research question three, we analyzed women’s responses to the abortion stigma scale. In their disclosures with people they were close to, women reported significantly more stigma in their disclosures about their abortions than in their disclosures about APR (*p* = 0.006). Additionally, women were more likely to have reported ever asking someone to keep their abortion a secret than their reversal (OR = 2.21), although the difference was not significant. To answer research question four, we analyzed women’s impressions of their partners’ preferences for abortion and APR. More women reported that their partners wanted the abortion (n = 12, 33%) than wanted the reversal (n = 8, 22%). Although only a few women reported that their partners did not want the abortion, this group of women reported more stigmatized disclosures about their abortions (M = 2.37, SD = 0.76) than did women whose partners wanted the abortion or who were ambivalent or not involved (M = 1.60, SD = 0.75) (t(34) = 2.11, *p *= 0.04) (Table 2). There was no significant difference in women’s disclosures about their APR experiences based on the partners’ opinions. Of the eight women who said that their partners wanted APR, seven described their decision-making as very or somewhat easy (88%), while only 54% (n = 15) of women whose partners did not want APR or were ambivalent described their decision-making as easy, although the difference was not significant. Only two women described their abortion decision-making as very or somewhat easy, including one woman who said her partner did not want the abortion and one woman whose partner did want the abortion.

**Table 2:** Demographic factors and medication abortion and abortion pill reversal stigma *p *< 0.05 was considered statistically significant APR: Abortion pill reversal

Demographics	Abortion			APR		
	N	Mean	*p*-value	N	Mean	*p*-value
Income						
Less than $90,000	29	1.87		29	1.49	
$90,000+	6	1.09	0.022	6	0.67	0.089
Religion						
Protestant or other Christian	18	1.51		18	0.99	
Other or no religion	17	1.98	0.074	17	1.74	0.040
Pregnancy Outcome						
Delivery	18	1.55		18	0.99	
Loss	7	1.90	0.340	7	2.14	0.015
Partner Preferences						
Partner did not want	5	2.37		11	1.11	
Partner wanted/ambivalent/uninvolved	31	1.60	0.042	25	1.41	0.44

To answer research question five, we examined factors associated with differences in women’s stigma scores. Overall, women who were higher income, Protestant or other Christian, and who delivered rather than having an abortion or miscarriage reported lower stigma scores. When sharing their disclosures about their abortions, women with a household income of $90,000+ reported less stigma (M = 1.09, SD = 0.52) than women making less than $90,000 (M = 1.87, SD = 0.76) (t(33) = 2.39, *p* = 0.022) (Table 2). Higher-income women also reported less stigma on average in their disclosures about their APRs (M = 0.67, SD = 0.47) than did women whose household income was less than $90,000 (M = 1.49, SD = 1.12), although the difference was not significant (t(33) = 1.75, *p* = 0.089). Non-Catholic Christian women experienced the lowest levels of stigma in their disclosures regarding their APR experiences (M = 0.99, SD = 0.80) (t(33) = 2.14, *p* = 0.040), as well as in their disclosures about their abortions, although the difference was not significant (t(33) = 1.85, *p* = 0.074).

Women who delivered a baby after undergoing APR reported lower stigma in relation to their disclosures about their APR experience (M = 0.99, SD = 0.91) than women who suffered a miscarriage or chose abortion (M = 2.14, SD = 1.16) (t(23) = 2.63, *p* = 0.015) (Table 2). For the pregnancy loss group (miscarriage and abortion), APR stigma was higher than abortion stigma, while women who delivered or were still pregnant reported more stigma in their abortion disclosures, although the differences were not significant.

## Discussion

Women’s decisions to seek medication abortion and APR are influenced by other people. Our exploratory analysis is the first of its kind to assess the influence that partners, family, and people whom women are closest with have in their decision-making to start a medication abortion and then immediately seek APR within 72 hours after taking mifepristone. Previous scholars have found that there is a diversity of reasons why women choose medication abortion, including selecting a process that feels more natural, avoiding a surgical procedure, offering more respect to the baby, allowing a more flexible schedule, and undergoing the abortion at home [14]. Our study adds to this scholarship by examining the influence of others in this decision-making process and the memorable messages received from others during their two different decision-making periods. Memorable messages are shown to influence health behavior [9], and our study highlights the ways in which different people and online messages from organizations impact women’s internalization of their decisions to seek medication abortion, and APR. Most of the women surveyed mentioned talking about their medication abortion decision with someone else (e.g., partner, family, friend, medical provider). Thus, the decision to have a medication abortion was not made in isolation but, rather, shaped by the memorable messages and opinions of other people, which other scholars have also found [17,25]. However, women’s disclosure patterns when seeking out information about APR tended to be different from their disclosures with others about the medication abortion decision. The APR decision is influenced by online messages that they independently seek out from Google after taking mifepristone. Since the decision to seek APR happens within 72 hours after taking mifepristone, the rapid change of heart suggests that women may not be fully informed or confident in their initial decision to seek medication abortion. Thus, women reported less stigma from others in their disclosures about APR than when talking about their medication abortions.

Other research on medical decision-making has found that patients typically integrate the information they receive from healthcare providers with information from other sources (e.g., partners, family, friends, internet) [26]. Some decision-making styles (e.g., relationship-embedded) place a significant emphasis on family members and their opinions regarding medical decisions [26], and family members are often an important site for memorable messages that impact behavior and self-concept [9]. When choosing medication abortion, women reported seeking the opinions of their partner and other family members first before choosing to take the mifepristone. Thus, these women adopted a relationship-embedded style [26] that considered others' opinions as a part of their own decision to take mifepristone. In contrast, when choosing APR, this decision was made more independently and was typically influenced by messages from the internet and after talking on the phone with healthcare professionals and medical providers who provided APR. In these instances, women adopted more of a medical-expert decision style, which aligns with traditional perspectives of the doctor-patient relationship [26]. Further, these memorable messages typically came from individuals who were older and of higher status, which are two common features found in the memorable messaging scholarship [9].

The number of women seeking medication abortion and APR is increasing [1,4]. Therefore, studying the messages and people who influence women’s decisions is important because a lack of personal autonomy in the decision-making process and limited emotional support from others have contributed to women’s feelings of regret after an abortion [17,18]. Like other scholars, we conclude that medication abortion decision-making is multidimensional and complex [14,17] and influenced by others who share memorable messages during women’s decision-making processes. Women’s decisions to choose medication abortion or APR are often not made flippantly, but rather wrought with dialectical tensions where women report feeling both unprepared and knowledgeable about their medication abortion decision, as well as a combination of relief and regret after having the medication abortion [15]. The women in this study further illustrate the complexities of medication abortion decision-making since all women sought APR within 72 hours (about three days) after taking mifepristone, and 21 of the 36 women contacted an organization to seek APR less than 12 hours after taking mifepristone. While some women are sure of their abortion decisions and do not experience prolonged regret, these findings point to the existence of a subset of women who experience a high degree of internal turmoil and immediate regret upon commencing the medication abortion process. Decision difficulty is also found to result from perceived pressure from the partner, positive feelings towards the pregnancy, and general difficulty with making decisions [26]; all of these variables were also factors associated with many of the women in our study.

This sample represents a group of women who began the medication abortion process and then almost immediately changed their minds. In a previous study, we found that some women undergoing medication abortion expressed feeling a need for more information and decision-making support, both from their abortion provider and their loved ones [15]. Similarly, a prior study of this same dataset found that overall, women reported poor communication with their abortion providers [16]. As new systems to distribute medication for abortion with little to no interaction between the patient and the medical professional providing the abortion become more mainstream, these models of care may be insufficient for some women. Our findings underscore the importance of a robust informed consent process with additional space and attention offered to women who may be experiencing high decisional difficulty or limited social support. Specifically, women should be provided with counseling and empowered with information on their options and alternatives [17].

Previous research on decision difficulty has found that partners and parents may have a considerable influence on the abortion decision and women’s feelings post-abortion [15,17]. Women’s thoughts and feelings regarding abortion decisions are typically associated with the decisional experiences of the involved male partner [25], and in our study women who reported that their partners wanted the medication abortion had lower abortion stigma scores. Similarly, a higher percentage of women whose partners wanted APR reported that choosing APR was easy when compared to women whose partners did not want APR or who were ambivalent or uninvolved in this decision.

Our finding that Protestant and other Christian women reported the lowest levels of stigma is notable because other scholars have found that Protestant women report elevated levels of abortion stigma compared to non-Christian women [23]. This significant difference may be due to our analysis having only a small sample of non-Christian women. Women’s pregnancy outcomes during the APR process may also affect stigma since women who had a live birth reported lower levels of stigma compared to women who had a pregnancy loss occur during the APR process. Research on the experiences of women who had initially desired an abortion but were unable to obtain one found that their emotions regarding their situations became more positive following the births of their babies [27].

There are several limitations within this initial exploratory analysis. First, data is self-reported; it is possible that some women were not comfortable disclosing certain information in the study. All women were over the age of 20 and the majority were White Christian women. Thus, our analysis should not be presumed as representative of the national population. Our specific aim was to examine person-level variation rather than draw generalizable conclusions. The most notable limitation is our small sample size. However, it is not surprising given that low response rates are typical of survey research studying abortion [28], and many women fall out of contact with the organization providing contacts to APR providers due to an eagerness from women to move past their experience with abortion and APR (personal communication, 2022). In an attempt to increase participation, we conducted multiple attempts via follow-up emails and text messages to contact eligible women, nonetheless, these attempts were futile. Consequently, another limitation of our study is that women with negative experiences with either abortion or APR may be underrepresented. Finally, some critics of APR state that there is no high-quality evidence from a randomized control trial to assess the effectiveness of progesterone as an APR protocol. However, the absence of clinical trials does not mean the treatment is ineffective [29]. In several clinical specialties, such as pediatrics, oncology, or the treatment of rare diseases, off-label drugs or treatments with limited high-quality evidence are often utilized and prescribed for these patients. Furthermore, APR’s biological effect has been demonstrated [5], and the use of supplemental progesterone in pregnancy has been established in the medical literature [11]. For a clinician to fail to offer APR to a pregnant woman who wants to preserve her pregnancy after taking mifepristone would be unethical [29]. Thus, the dearth of scholarship on this topic and the subpopulation of women provides credence for our study to establish an initial understanding and baseline needed for future scholarship exploring the voices of these muted and marginalized women.

## Conclusions

In conclusion, medication abortion and APR are increasing in frequency of use given the recent Supreme Court decision in *Dobbs vs. Jackson Women’s Health Organization* (2022). This legal situation not only impacts women’s use but also changes the larger societal discourses surrounding medication abortion and the ways in which women talk about their medical decisions with family and friends. Health providers not only need to be equipped to provide comprehensive informative counseling to pregnant women by seeking informed consent but also to help these women navigate future conversations with support networks regarding their personal decisions. Our findings shed some initial insight into the ways in which some women currently navigate these conversations with others and the memorable messages received during their medication abortion and APR decisions. More research on this growing population is needed to provide quality perinatal care for women and help them exercise their personal choices for medication abortion and/or APR. Two potential avenues for future scholarship include surveying a larger sample of women in order to make generalizable claims, as well as longitudinally examining women’s abortion and APR experiences over time.
